# The Role of Carbohydrates in the Lipopolysaccharide (LPS)/Toll-Like Receptor 4 (TLR4) Signalling

**DOI:** 10.3390/ijms18112318

**Published:** 2017-11-03

**Authors:** Florent Cochet, Francesco Peri

**Affiliations:** Department of Biotechnology and Biosciences, University of Milano Bicocca, Piazza della Scienza, 2, 20126 Milano, Italy; f.cochet@campus.unimib.it

**Keywords:** lipopolysaccharide, TLR4 (Toll-like receptor 4), MD-2 (myeloid differentiation factor 2), Kdo (3-deoxy-d-manno-octulosonic acid)

## Abstract

The interactions between sugar-containing molecules from the bacteria cell wall and pattern recognition receptors (PRR) on the plasma membrane or cytosol of specialized host cells are the first molecular events required for the activation of higher animal’s immune response and inflammation. This review focuses on the role of carbohydrates of bacterial endotoxin (lipopolysaccharide, LPS, lipooligosaccharide, LOS, and lipid A), in the interaction with the host Toll-like receptor 4/myeloid differentiation factor 2 (TLR4/MD-2) complex. The lipid chains and the phosphorylated disaccharide core of lipid A moiety are responsible for the TLR4 agonist action of LPS, and the specific interaction between MD-2, TLR4, and lipid A are key to the formation of the activated complex (TLR4/MD-2/LPS)_2_, which starts intracellular signalling leading to nuclear factors activation and to production of inflammatory cytokines. Subtle chemical variations in the lipid and sugar parts of lipid A cause dramatic changes in endotoxin activity and are also responsible for the switch from TLR4 agonism to antagonism. While the lipid A pharmacophore has been studied in detail and its structure-activity relationship is known, the contribution of core saccharides 3-deoxy-d-manno-octulosonic acid (Kdo) and heptosyl-2-keto-3-deoxy-octulosonate (Hep) to TLR4/MD-2 binding and activation by LPS and LOS has been investigated less extensively. This review focuses on the role of lipid A, but also of Kdo and Hep sugars in LPS/TLR4 signalling.

## 1. Innate Immunity Response in Higher Organisms to Pathogen-Associated Molecular Patterns

Mammalian innate immunity relies on a family of pattern recognition receptors (PRRs) to detect conserved microbial molecules, termed pathogen-associated molecular patterns (PAMPs) [1,2]. PRRs engagement by microbial PAMPs activates inflammatory pathways, such as the nuclear factor kappa B (NF-κB) and interferon regulatory factor (IRF) signalling for cytokine transcription and the clearance of the infections. The PRR family includes Toll-like receptor (TLR), C-type lectin receptor (CLR), RIG-I-like receptor (RLR), AIM2-like receptor (ALR), and NOD-like receptors (NLR) containing a Nucleotide-binding domain (NBD) and a Leucine-rich Repeat (LRR) domain. TLR and CLR are localized on the plasma or endosomal membranes, while RLR, ALR, and NLR are cytoplasmic [3,4]. The host receptorial system is redundant in the sense that a collection of structurally diverse PRRs recognizes the same bacterial cell wall component. PRRs then induce various signalling pathways that depend on structurally diverse signalling components [5].

Some of the most inflammatory PAMPs, such as lipopolysaccharide (LPS), lipoproteins, peptidoglycan (PGN), and flagellin, are molecules derived from the bacterial cell wall and are recognized, respectively, by specific PRRs.

**Figure 1:**
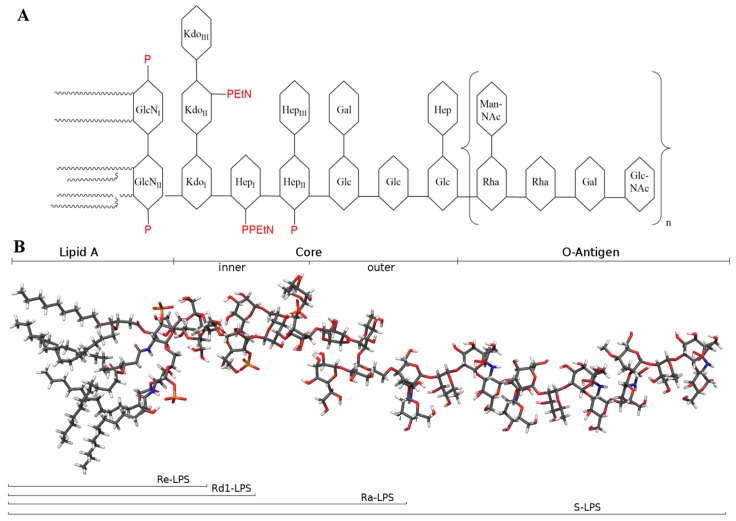
*E. coli* lipopolysaccharide (LPS): smooth-lipopolysaccharide (S-LPS) is composed by lipid A, core and O-antigens; truncated rough-LPS (R-LPS) are named Ra, Rd1, and Re depending on the number of sugar units of the core. (**A**) Schematic representation (**B**) Chemical structure

Organic chemistry synthesis of chemically homogeneous molecules greatly contributed to the unequivocal determination of the minimal and chemically defined structure that is essential for the immunostimulating actions of LPS [6,7,8]. LPS is composed of three distinct domains (Figure 1) that are covalently linked to each other, which are genetically, biosynthetically, biologically, and chemically distinct: a glycolipid portion termed lipid A, a glycan, and between them a core oligosaccharide.

The glycan usually is the O-specific polysaccharide (or O-antigen). The membrane-anchoring portion called lipid A is also the biologically active unit (endotoxic principle). The first chemically synthesised lipid A [9], according to the newly elucidated structure, exhibited full activity described for LPS endotoxin.

In the classical nomenclature [10] (Figure 1) LPS is identified by the appearance of the corresponding bacterial colony surface: smooth colonies express complete LPS that has been accordingly termed smooth-lipopolysaccharide (S-LPS). Mutants that express LPS lacking O-antigens form colonies with rough appearance, so that the corresponding truncated LPS variants have been named rough-LPS (R-LPS). However, not all of the mutants lose all O-antigen, so that there are three types of R-LPS (Figure 1). Ra-LPS mutant is composed of the Lipid A and the complete oligosaccharide core, Rd1-LPS mutant is formed by Lipid A and the inner core and the smaller variant, Re-LPS, also called deep rough mutant, only contains two or three Kdo units linked to Lipid A [11,12].

Complete LPS (S-LPS), and its truncated rough variants (R-LPS) including lipid A (Figure 1) are generally defined as endotoxin and have potent pro-inflammatory and immunostimulating action.

Free LPS released from gram-negative cell wall, in form of single molecules or aggregates, is detected by the Toll-like Receptor 4 (TLR4), which is mainly expressed on the surface of haematopoietic cells including monocytes, dendritic cells, and macrophages [11].

A recent review by Kieser and Kagan [5] presents an updated, more complex picture of the LPS recognition by innate immunity cells, suggesting that several membrane and cytosolic receptors are activated by LPS variants. Membrane receptor Brain-specific angiogenesis inhibitor 1 (BAI1) also detects LPS at the bacteria surface and, after promoting phagocytosis, triggers reactive oxygen species (ROS) production and the induction of inflammation. Moreover, LPS that has reached the cytosol is recognized by caspases, initiating formation of the non-canonical inflammasome [12].

From a molecular point of view, glycoconjugate/protein interactions are the key events of activation of innate immunity PRRs, including cytosolic caspases, triggering cytokine production and inflammation. In this review, we will focus on molecular details of sugar-protein interactions in the case of TLR4/MD-2/LPS signalling, the most important and studied pathway in innate immunity and inflammation.

## 2. Physicochemical Properties of LPS and Molecular Mechanism of LPS/TLR4 Signalling

The amphiphilic character of endotoxin chemical variants (S-LPS, R-LPS or lipid A), results in the formation of micelles in aqueous environment above their critical micellar concentration (CMC) [13]. CMC values between 10^−8^ M and 10^−7^ M for deep rough mutant (Re-LPS) [14,15], and between 1.3 and 1.6 μM for *E. coli* S-LPS [16], were reported. In balanced salts solutions containing physiologic extracellular concentrations of Mg^2+^ and Ca^2+^, CMC values of 1 nM or lower are likely [17,18].

From these data and from the fact that LPS aggregates are usually highly stable, aggregated forms of LPS should predominate in the concentration range that is relevant for biological responses. In physiological fluids, LPS aggregates were also found as membrane “blebs”, which are constitutively released from growing Gram-negative bacteria. Transmission electron microscopy (TEM) revealed that blebs exist predominantly as vesicles with an average size of 40–80 nm [19].

**Figure 2:**
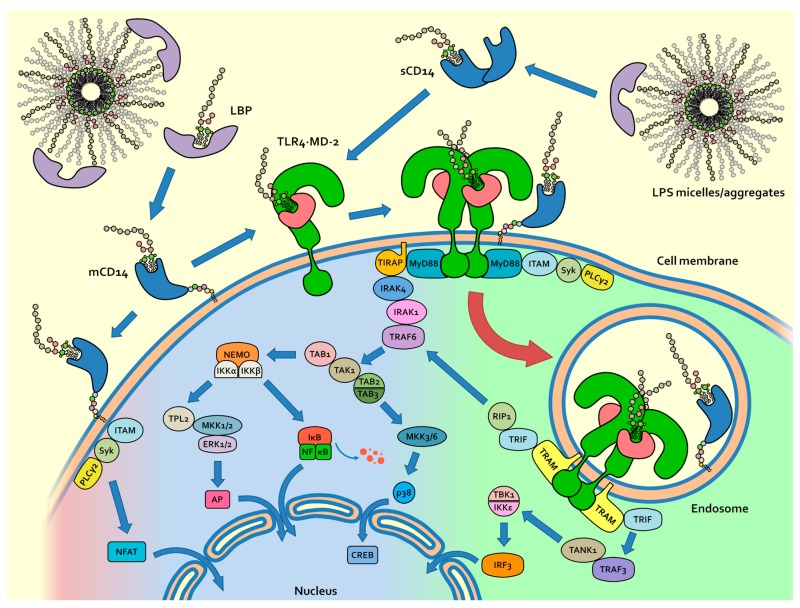
TLR4 activation and signalling from cell membrane and from endosomes: MyD88 and TRIF-dependent pathways are distinct signal pathways leading to cytokine production.

The current view of mammalian endotoxin sensing and signalling (Figure 2) is that it is initiated by the lipid-binding protein (LBP), which is able to extract LPS monomer from the aggregates and to transfer it to the cluster of differentiation 14 (CD14) [20,21]. CD14, then, transfer the LPS monomer to Myeloid Differentiation factor 2 (MD-2) adaptor in the final hexamer complex (TLR4/MD-2/LPS)_2_ (Figure 2) [22,23,24,25]. This unidirectional flow of LPS from LBP to TLR4/MD-2 is explained by the increasing affinity of each LPS receptor for its ligand [26]. TLR4 crosslinking through the formation of the (TLR4/MD-2/LPS)_2_ complex is the first step in the inflammatory process. The CD14 receptor, in the membrane-bound form, promotes the formation of hexamer complex (TLR4/MD-2/LPS)_2_ by binding monomeric LPS and transferring it to MD-2, thus initiating the Myeloid differentiation primary response 88 (MyD88)-dependent intracellular signalling [26,27]. CD14 also plays a fundamental role in the endocytosis of (TLR4/MD-2/LPS)_2_ and in subsequent intracellular signalling based on triffosome formation [28,29,30,31,32].

In general, the activation of TLRs by their ligands is the first molecular event of innate immunity, preceding and triggering cytokine production, inflammation and adaptive immune response [33]. From a pharmacological point of view, TLR4 stimulation by non-toxic LPS/lipid A variants is considered as an innovative approach towards potent and selective immunostimulants to be used as vaccine adjuvants and in tumor immunotherapy [34,35]. On the other hand, natural LPS variants or synthetic molecules that inhibit the formation of (TLR4/MD-2/LPS)_2_ hexamer complex by competing with LPS or other agonists for TLR4/MD-2 and/or CD14 binding are interesting drug candidates targeting diseases that are caused by excessive TLR4 activation by bacterial LPS (sepsis and septic shock) and by endogenous molecules (inflammatory and autoimmune diseases) [36].

The knowledge of the molecular aspects of LPS recognition by CD14 and TLR4/ MD-2 is essential to understand the different TLR4-mediated responses to different LPS/lipid A variants or chemotypes, whose activity varies from toxic (TLR4 agonists), to non-toxic and even LPS with endotoxin-neutralizing activity, corresponding to TLR4 antagonism. The same molecular mechanisms play a fundamental role in innate memory and tolerance to LPS [37]. Finally, the rational design of TLR4 agonists and antagonists as drug candidates relies on the precise knowledge of the molecular aspects of the interaction between lipid A and CD14 and/or MD-2 receptors.

## 3. Structure-Activity Relationship in Natural and Synthetic Lipid A Variants

The chemical pattern consisting in the disaccharide β(1–6) glucosamine core with two phosphates at C1 and C4′ positions and branched or linear fatty acid chains linked to C2, C3, C2′, and C3′ positions is highly conserved in lipid As of different bacterial species. It is the pharmacophore associated to endotoxic activity. This motif can be found in the natural and synthetic lipid A variants that bind and activate TLR4, thus behaving as agonists. The structural variations of lipid A found in different bacterial species (different acylation patterns, variation in the chemical structure and length of fatty acid chains, covalent modification of phosphate groups), are associated to different biological activity.

**Figure 3:**
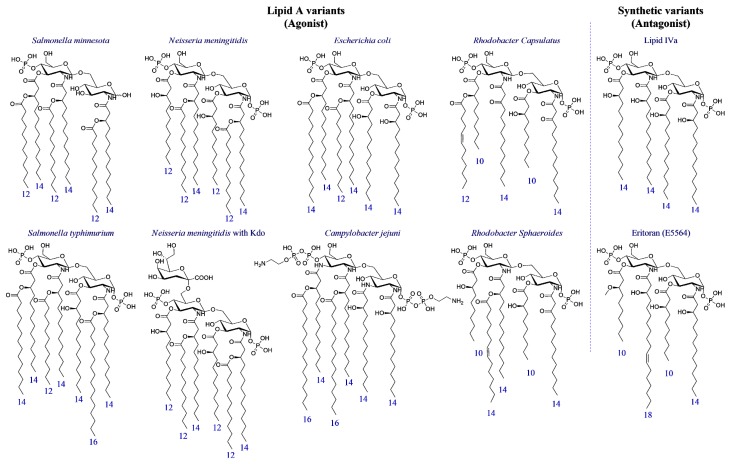
Natural lipid A from *Salmonella minnesota*, *Salmonella typhimurium*, *Neisseria meningitides* (with and without Kdo), *Escherichia coli*, *Campylobacter jejuni*, *Rhodobacter capsulatus* and *Rhodobacter sphaeroides*; the biosynthetic lipid A precursor: Lipid IVa and the synthetic molecule Eritoran. The numbers of carbon atoms of each fatty acid chain are displayed in blue.

Natural Lipid A variants produced by *E. coli*, *Neisseria meningitidis*, *Campylobacter jejuni*, *Salmonella minnesota* or *tiphimurium*, *Rhodobacter capsulatus* or *sphaeroides* species, as well as biosynthetic precursor lipid IVa and synthetic analogue Eritoran [38] (Figure 3), provided complete information on structure-activity relationship (SAR) of this class of compounds.

Lipid A variants differ in the acylation pattern, that is the location of fatty acid chains on the disaccharide: 4 + 3 (corresponding to four chains attached to the non-reducing and three chains in the reducing glucosamine) in *S. tiphimurium,* 4 + 2 in *E. coli*, *C. jejuni* and *S. minnesota*, 3 + 3 in *N. meningitidis*, 3 + 2 in *R. capsulatus* and *sphaeroides*, 2 + 2 in Lipid IVa. Synthetic Eritoran presents a 2 + 2 arrangement as well. Lipid As also presents different composition and length of chains (*E. coli* contains mainly C_14_ chains while *N. meningitides* contains mainly C_12_ chains).

Crucial information on SAR of lipid A variants has been collected by Boons and co-workers by means of fully synthetic derivatives [39]. This study highlighted that synthetic lipid A from *N. meningitidis* was significantly more potent than *E. coli* lipid A. According to these results, the 3 + 3 arrangement of fatty acid chains in *N. meningitidis* is associated with a stronger endotoxic activity than the 4 + 2 disposition found in *E. coli*. Moreover, shorter fatty acids chains with 12 instead of 4 carbon atoms are associated to a higher potency in TLR4 stimulation (1–2 orders of magnitude) [39].

It is quite unusual to observe unsaturated fatty acids in lipid A, but it has been reported in *Rhodobacter sphaeroides* and *R. capsulatus* LPS and in the *Enterobacteriaceae* family when they grow at low temperature. *Campylobacter jejuni* LPS is an exception, in which GlcN are replaced by 2,3-diamino-2,3-dideoxy-d-glucose (GlcN3N) [40] (Figure 3).

In some natural lipid A variants, phosphates in positions C1 and C4′ are covalently functionalized with phosphate, ethanolamine, ethanolamine phosphate, ethanolamine di-phosphate (*C. jejuni*), and sugars as glycosylamine, 4-amino-4-deoxy-l-arabinopyranose and d-arabinofuranose [41].

In general, while the presence of six fatty acid (FA) chains is associated with endotoxic action (TLR4 agonism), variants with 7 FA, as *Salmonella typhimurium*, or with less than 6 FA, as in lipid IVa or in the synthetic derivative Eritoran, are on the contrary associated to non-toxic or even anti-endotoxic activity. The anti-endotoxic activity is defined as the competitive antagonist action of lipid A or LPS variants that, when co-administered with endotoxin, neutralize its TLR4 activating effect. A general pharmacophore can be associated to antagonism, consisting of four or five FA that are linked to a disaccharide core bearing in C1 and C4′ positions in one or two negatively charged groups (phosphates or their bioisosteres) [42].

Monosaccharide-based TLR4 modulators allowed for extending the agonism/antagonism rules to the monosaccharide scaffold. The 3:1 ratio between lipid chains and phosphate groups present in lipid A seem an important prerequisite to have agonistic activity both in lipid A variants and in monosaccharides derivatives [43]. Synthetic monosaccharides derivatives with one or two phosphates and two to four fatty acid chains showed in some cases potent TLR4 antagonism in cells and in vivo [44,45,46,47]. In mono- and di-saccharides scaffolds, the phosphate groups have been substituted with bioisosteric, negatively charged carboxylates, and showed similar activities [48,49,50,51].

The elucidation of new lipid A chemical structures deriving from bacteria species is a field in continuous evolution, and SAR in new lipid A types will provide important information for designing new synthetic TLR4 modulators [41,52].

In addition, some natural or synthetic molecules having a chemical structure that is unrelated to lipid A can bind to MD-2 and activate TLR4. The natural compounds Taxol and some opioids activate TLR4 [53,54]. Synthetic pyrimido[5,4-b]indoles [55] and 4-substituted aminoquinazolines [56] were shown to specifically activate TLR4 in a MD-2 dependent and CD14 independent manner, in both mouse and human cells. Synthetic compounds with a linear scaffold instead of glucosamine disaccharide such as ER-112022 [57] are active as TLR4 agonist, thus showing that the disaccharide connecting the two anionic phosphates can be substituted by chemical spacers of different chemical structure and with increased conformational mobility. More recently, new classes of non-LPS-like small molecules, the neoseptins [58,59], Euodenine A and analogues [60], have been reported to be MD-2-dependent TLR4 agonists [5,59,61,62,63].

## 4. Role of Sugar/Protein and Lipid/Protein Interactions in the Formation and Stability of TLR4/MD-2/Endotoxin Complex

**Figure 4:**
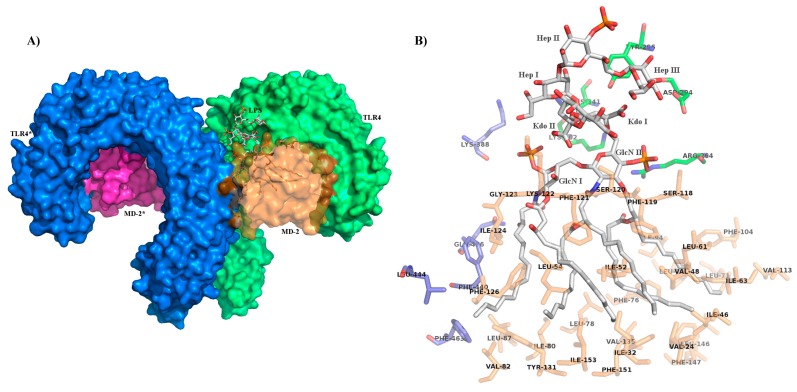
Picture of the interactions between LPS, (TLR4/MD-2) complexes (**A**) (TLR4/MD-2/LPS)_2_ signalling complex; (**B**) *E. coli* Lipid A with inner-core and its interactions with MD-2 (residues in orange) and TLR4 (residues in green) of the same complex and with the second TLR4 molecule, TLR4* (blue), as observed by crystallography (PDBID: 3FXI). LPS is displayed in gray for carbon atoms, red for oxygen, blue for nitrogen and dark orange for phosphate.

The crystal structures of the hexamer complex (TLR4/MD-2/LPS)_2_, in which two TLR4 are crosslinked [22] (Figure 4A), has revealed that binding of LPS (Ra-LPS from *E. coli*) to MD-2 induces agonist-dependent contacts with the C-terminal domain of the second TLR4 molecule of the hexamer (TLR4*). This binding mode is typical of agonists since it is responsible for TLR4 dimerization and activation. Five of the six aliphatic chains of lipid A moiety are deeply inserted into the hydrophobic pocket of MD-2. The FA chain linked to C2 of GlcN I protrudes from MD-2 binding cavity forming, with MD-2 residues (V82, M85, L87, I124, and F126), a binding interface that interacts with hydrophobic residues on the surface of TLR4* (mainly F440*, L444*, and F463*), thus promoting the assembly of the activated hexamer. Additional hydrophilic interactions are also present and involve lipid A’s hydroxyls and phosphates groups. These groups interact with MD-2 and TLR4* residues and stabilize the hexameric complex (Figure 4B: C1 phosphate interacts with K388*, K341 and K362 for the two TLR4 as well as K122 for MD-2; C4′ phosphate is in contact with R264, K362 for TLR4, and K58, S118 for MD-2. Moreover, C4-hydroxyl group of the GlcN I interact with K122 residue).

Upon lipid A binding, MD-2 experiences a local conformational change, which involves the side chain of F126 and its immediate neighbours [64]. The interaction between lipid A and TLR4/MD-2 has been investigated by NMR in solution and suggests a dynamic role of F126. This phenylalanine residue is located at the rim of the LPS binding cavity of MD-2, and acts as a conformational switch allowing, or not, the formation of activated hexamer [65].

**Figure 5:**
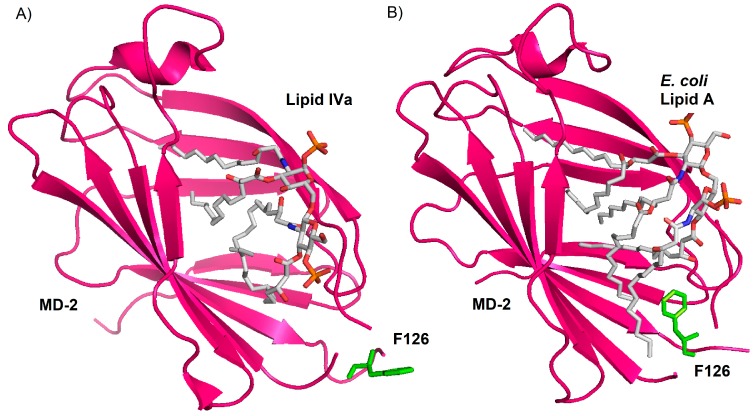
(**A**) Cristal structure of human MD-2 with Lipid IVa (PDBID: 2E59); (**B**) Cristal structure of human MD-2 with *E. coli* Lipid A (PDBID: 3FXI). Human MD-2 is displayed in purple as ribbon, F126 are displayed in green as sticks and Lipid IVa and Lipid A are displayed according to the color of the atoms as sticks.

In contrast to hexa-acylated lipid A forms, tetra-acylated lipid IVa is a weak agonist in murine TLR4/MD-2, but is an antagonist in human TLR4/MD-2. The crystal structures of human and mouse TLR4/MD-2 in the complex with tetra-acylated lipid IVa provided the structural basis of this species-specific agonistic or antagonistic activities [66,67]. In human MD-2 complex with lipid IVa, the GlcN-P backbone of lipid IVa was shifted upward and rotated by about 180° in comparison to hexa-acylated LPS [64,68] (Figure 5).

This binding mode is not productive and it does not promote the formation of the signalling complex, it is typical of antagonists. Eritoran binds both human and mouse MD-2 with the disaccharide core rotated of 180° respect to agonist lipid A [69]. The interactions of hydrophilic and hydrophobic groups of lipid A with MD-2 and TLR4* have been studied in order to understand the affinity of lipid A variants with the receptor complex, and to explain the switch from agonism to antagonism [61,70,71]. The ligand-based design of new synthetic TLR4 agonist and antagonists has been guided so far by the necessity to reproduce the most important supramolecular interactions among lipid A, MD-2, TLR4, and TLR4* [46,72,73,74].

## 5. Role of the Sugar Kdo in the TLR4 Interaction of Natural and Synthetic LPS Variants

The LPS inner core (Figure 1) is typically composed by two or three Kdo units, linked via an α-(2 → 6) linkage to the distal GlcN of the lipid A backbone and three units of Hep. The outer core is composed by more common monosaccharides, such as Glucose (Glc) and galactose (Gal) [75]. The inner core structure is reasonably well conserved (Figure 1) [76], and it is therefore an epitope that is capable of inducing a specific antibody to cross-react with the LPS of many different strains of Gram-negative LPS. Immuno-response directed against the inner core has been exploited in vaccines to elicit widely cross-reactive antibodies in human sera [77] and in the production of a monoclonal antibody that is able to bind to a wide range of *E. coli* and *Salmonella* LPSs [78,79].

It has long been thought that the inflammatory properties of S-LPS and R-LPS reside only in the lipid A moiety [6,80], and that the nature and number of core saccharides do not have major impact on modulating endotoxicity [81].

From a structural point of view, the inner core sugars, three units of 3-deoxy-d-manno-2-octulosonic acid (Kdo I, II, III) and three units of heptosyl-2-keto-3-deoxy-octulosonate (Hep I, II, III) seem to contribute only marginally to the interactions with receptors. However, Kdo I, Hep I, and Hep III are interacting with the TLR4 residues Y296, K341, and D294, respectively (Figure 4). The fact that the inner core sugar interacts only with TLR4, and not with MD-2 and TLR4* as lipid A, confirms that the presence of the inner core sugars is not essential for the endotoxic activity of LPS [9,80], but also suggests that such additional interaction with TLR4 could be important for increasing LPS binding affinity and specificity to the MD-2/TLR4 heterodimer.

Additionally, recent studies have indicated that Kdo moieties of the inner core are likely to contribute to inflammatory responses [7,82,83,84,85,86,87]. It has also been proved that the *N. meningitidis* Ra-LPS is a potent agonist of MyD88-dependent and independent cytokines [88].

Several other studies have indicated that Kdo moieties of LPS and/or Ra-LPS importantly contribute to inflammatory responses and to TLR4 activation and signalling [85,89].

Boons and co-workers used chemically pure synthetic lipid A variants, with or without Kdo glycosylation [39], and showed that the activity of *N. meningitidis* Re-LPS in eliciting TNF-α and IFN-β production in murine macrophages was significantly higher compared to the synthetic *N. meningitidis* lipid A. As well, the induction of TNF-α was dramatically decreased, to levels like those of the lipid A, when penta-acylated Ra-LPS was subjected to mild acid hydrolysis (pH 4.3), thus separating lipid A from Kdo.

The loss of the two Kdo from penta-acylated meningococcal Re-LPS resulted in a dramatic attenuation in biologic activity [90]. It is worth noting that *N. meningitidis* Re-LPS is recognized by the CD14, TLR4/MD-2 receptors of both human and murine cells. It suggests that the two Kdo of Re-LPS are not determinant for species-specific differentiation, noted for other LPS structures.

Zughaier et al. compared the responses of human monocyte derived dendritic cell (MDDC) to *Neisseria meningitidis* LPS, Re-LPS, and lipid A [85]. These three endotoxin variants were obtained from genetically modified bacteria to afford highly controlled serotypes [91,92,93]. It was found that similar to LPS, Re-LPS induced MDDC maturation and significantly up-regulated the expression of CD80 and CD86 co-stimulatory molecules, the MDDC maturation marker, CD83 and HLADR (Human Leukocyte Antigen D Related). On the contrary, lipid A was much less active in inducing the production of the same co-stimulatory molecules. Again, these data suggest that the presence of the two Kdo in Re-LPS greatly increases the activity of lipid A and the authors concluded that Re-LPS is the minimal structure required to induce optimal MDDC maturation and activation.

The same authors produced different endotoxin variants in *N. meningitidis* and compared their activities in terms of cytokine release (TNF-α) and inflammatory mediators (nitric oxide) in macrophages. By a combination of genetic modification and mild acidic hydrolysis of S-LPS they obtained Rd1-LPS, Re-LPS, and lipid A [94,95,96]. They observed that Rd1-LPS and Re-LPS have similar activities while lipid A is ~10-fold less active. These data suggest that, while Kdo presence is necessary to optimal TLR4 agonism, the Hep units are dispensable [84]. Boons et al. examined the activity of a range of chemically defined, synthetic Lipid As [39,97,98] that were derived from *E. coli*, *S. typhimurium*, *S. minnesota*, and *N. meningitidis* bacterial strains on mouse macrophages and dendritic cells. It was found that the *N. meningitidis* Re-LPS was more potent than the Re-LPS from *E. coli*, which, in turn, were significantly more potent that the synthetic *E. coli* and *N. meningitidis* Lipid A [99].

In this case too, the two Kdo units linked to *N. meningitidis* lipid A were essential to TLR4 activity, even more important than having the two phosphate groups. Monophosphorylated *N. meningitidis* Lipid A was only slightly less active than the diphosphorylated *N. meningitidis* Lipid A, and *N. meningitidis* Re-LPS structures with variable 1 or 4’ phosphorylation were equal agonists. [100].

Muroi and Tanamoto observed similar trend in the case of *Salmonella* endotoxins activity on human THP-1 cells and TLR4-transfected HEK cells. In this case, the order of endotoxic activity was also S-LPS ≈ Re-LPS >> Lipid A. [82].

Schromm et al. [101] found that the number, nature, and location of negatively charged Kdo monosaccharide units modulate the molecular conformation of *E. coli* LPS and Lipid A, and that conformation is tightly linked to endotoxicity. Recently, synthetic Re-LPS (containing two or three Kdo) was found to have an enhanced agonist activity compared to Lipid A linked to one Kdo and Lipid A alone [7,42]. A decreased number or lack of Kdo and hydroxymyristic acid is proposed as main contributors to low endotoxic activity of *Leptospira interrogans* [102], *Francisella tularensis* [103], *Legionella pneumophila* [104], and different *Rhizobium* species LPSs [105].

From a structural point of view, the negatively charged Kdo sugars of LPS may facilitate the binding to leucine-rich repeats (LRR) of TLR4 (most likely to residues 190 to 194 that contain positively charged amino acids [106]) The presence of Kdo moiety plays also a fundamental role in the conformational change of the glucosamine backbone [101]. When linked to Kdo, an upward shift of GlcN I was observed (~4 Å) [22]. This shifted conformation permits to have an additional space for R2 and R3 acyl chains and bring phosphate group closer to TLR4 protein [107].

## 6. Conclusions and Future Perspectives

While the polysaccharide O-chain seems dispensable to TLR4 activation and signalling, sugars of the core oligosaccharide play a significant role in TLR4 activation. Kdo is important for agonist activity: fully synthetic lipid A containing Kdo units (also called Re-LPS) are always more active than their counterparts lacking Kdo [39]. This increase in the agonist activity is somehow not paralleled by the number of interactions between the Kdo units and MD-2/TLR4/TLR4*, as observed by crystallography: just one interaction per Kdo unit. It should however be considered that the number of interactions actually observed with X-ray may not be exhaustive, since Kdo and Hep carbohydrates protrude from MD-2 binding site, the high rotational freedom of core oligosaccharide can hamper the observation of the interactions with the TLR4/MD-2 complex. The Kdo role in TLR4 antagonism has not, or has only partially been investigated. In analogy with agonists, also antagonists should benefit from additive interaction of Kdo units with TLR4/MD-2 complex. Thanks to the production of lipid A variants with different number of Kdo units linked by chemical [7] or enzymatic [108] processes, it can be assessed that Re-LPS is the minimal chemical motif required for the maximal activation of (TLR4/MD-2/LPS)_2_ complex.

The synthesis of lipid A mimetics, bearing one to three Kdo units linked with non-natural bonds resistant to enzymatic hydrolysis, would provide a new generation of TLR4 antagonists that should improve potency and specificity.

Another important parameter that determines the efficiency of agonist and antagonist presentation to the TLR4/MD-2 receptor complex is the tendency of the ligands to form supramolecular aggregates in solution. The amphiphilic character of natural lipid As and synthetic lipid A analogues favors the formation of aggregates in solution. The stability of aggregates determines, in turn, the affinity of the ligands for LBP, the first receptor of the extracellular LPS/TLR4 signal pathway. In this context, the addition of hydrophilic sugar moieties to lipid A analogues could improve water solubility, increase the CMC values of molecules, and favor interaction with LBP.

In addition, the role of Kdo and Hep sugars in the binding with LBP and CD14 receptors has still to be investigated. Due to the important function of these two LPS-binding proteins in determining the efficiency of agonist or antagonist presentation to the final TLR4/MD-2 complex, an increase in affinity due to the presence of additive sugars could greatly influence the type and intensity of TLR4 response. CD14 has a leading role in the process of endocytosis of TLR4/MD-2 complex ending by intracellular signalling through the TRAM/TRIF complex formation. The differential affinity of different LPS forms for CD14 (complete S-LPS versus R-LPS lacking the O-chain) was clearly evidenced.

This review presents some examples in which the presence of Kdo in natural or synthetic lipid A variants (mono- or di-saccharides) improves the TLR4 activity if compared to simple lipid A or complete LPS with O-chain. The synthesis and the biological characterization of lipid A analogues glycosylated with Kdo and/or Hep (or mimetics of these sugars) is still largely unexplored.

We suggest that glycosylated lipid A analogues can provide in the next future a new generation of synthetic molecules to be developed as drugs targeting TLR4 signal.

## Abbreviations

AIM2	Interferon-inducible protein (Absent In Melanoma 2)
ALR	AIM2-Like Receptor
CD14	Cluster of Differentiation 14
CD80	Cluster of Differentiation 80
CD83	Cluster of Differentiation 83
CD86	Cluster of Differentiation 86
CLR	C-type Lectin Receptor
CMC	Critical Micellar Concentration
FA	Fatty acid
Gal	Galactose
Glc	Glucose
GlcN	Glucosamine
GlcN3N	2,3-diamino-2,3-dideoxy-d-glucose
GlcNAc	*N*-Acetyl-Glucosamine
GPI	Glycosylphosphatidylinositol
HEK	Human Embryonic Kidney Cell 293
Hep	heptosyl-2-keto-3-deoxy-octulosonate
HLADR	Human Leukocyte Antigen D Related
IL	Interleukin
INF	Interferon
IRF	Interferon Regulatory Factor
IRF3	Interferon Regulatory Factor 3
Kdo	3-Deoxy-d-manno-oct-2-ulosonic acid
LBP	Lipid Binding Protein
LOS	Lipooligosaccharide (Hep3-Kdo2-lipid A)
LPS	Lipopolysaccharide
LRR	Leucine-Rich Repeat
MC	Mast Cells
MD-2	Myeloid Differentiation factor 2
MDDC	Human Monocyte Derived Dendritic Cell
MurNAc	*N*-Acetyl-Muramic acid
MyD88	Myeloid differentiation primary response gene 88
NBD	Nucleotide-Binding Domain
NF-κB	Nuclear Factor-kappa B
NLR	NOD-Like Receptors
NMR	Nuclear Magnetic Resonance
NOD 1 & 2	Nucleotide-binding Oligomerization Domain receptor 1 & 2
PAMP	Pathogen Associated Molecular Pattern
PGN	Peptidoglycan
PRR	Pattern Recognition Receptor
Ra-LPS	Lipid A + core
Re-LPS	(Kdo)2-Lipid A
RIG-1	Retinoic Acid Inducible Gene 1
R-LPS	Rough LPS
RLR	RIG-I-Like Receptor
SAR	Structure Activity Relationship
S-LPS	Smooth LPS
TANK	TRAF family member-associated NF-κ-B activator
TBK1	TANK-Binding Kinase 1 (Serine/Threonine-protein Kinase TBK1)
TEM	Transmission Electron Microscopy
THP-1	Human Leukemic Monocyte Cell Line
TIR	Toll/interleukin-1 receptor
TLR4	Toll Like Receptor 4
TNF	Tumor Necrosis Factor
TRAM	TNF Receptor Associated Modulator
TRIF	TIR-domain-containing adapter-inducing interferon-β
